# Cloning and Molecular Characterization of an Alpha-Glucosidase (MalH) from the Halophilic Archaeon *Haloquadratum walsbyi*

**DOI:** 10.3390/life7040046

**Published:** 2017-11-21

**Authors:** Mara F. Cuebas-Irizarry, Ricardo A. Irizarry-Caro, Carol López-Morales, Keyla M. Badillo-Rivera, Carlos M. Rodríguez-Minguela, Rafael Montalvo-Rodríguez

**Affiliations:** 1 Biology Department, Box 9000, University of Puerto Rico, Mayagüez, PR 00681, USA; mara.cuebas@upr.edu (M.F.C.-I.); c_lopez_pr@yahoo.com (C.L.-M.); carlos.rodriguez66@upr.edu (C.M.R.-M.); 2 Department of Immunology, University of Texas Southwestern Medical Center, Dallas, TX 75390, USA; ricardo.irizarry-caro@utsouthwestern.edu; 3 Genetics Department, School of Medicine, Stanford University, Stanford, CA 94305, USA; kbadillo@stanford.edu

**Keywords:** alpha-glucosidase, halophilic Archaea, *Haloquadratum*, J0101

## Abstract

We report the heterologous expression and molecular characterization of the first extremely halophilic alpha-glucosidase (EC 3.2.1.20) from the archaeon *Haloquadratum walsbyi*. A 2349 bp region (*Hqrw_2071*) from the *Hqr. walsbyi* C23 annotated genome was PCR-amplified and the resulting amplicon ligated into plasmid pET28b(+), expressed in *E. coli* Rosetta cells, and the resulting protein purified by Ni-NTA affinity chromatography. The recombinant protein showed an estimated molecular mass of 87 kDa, consistent with the expected value of the annotated protein, and an optimal activity for the hydrolysis of α-PNPG was detected at 40 °C, and at pH 6.0. Enzyme activity values were the highest in the presence of 3 M NaCl or 3–4 M KCl. However, specific activity values were two-fold higher in the presence of 3–4 M KCl when compared to NaCl suggesting a cytoplasmic localization. Phylogenetic analyses, with respect to other alpha-glucosidases from members of the class Halobacteria, showed that the *Hqr. walsbyi* MalH was most similar (up to 41%) to alpha-glucosidases and alpha-xylosidases of *Halorubrum*. Moreover, computational analyses for the detection of functional domains, active and catalytic sites, as well as 3D structural predictions revealed a close relationship with an *E. coli* YicI-like alpha-xylosidase of the GH31 family. However, the purified enzyme did not show alpha-xylosidase activity. This narrower substrate range indicates a discrepancy with annotations from different databases and the possibility of specific substrate adaptations of halophilic glucosidases due to high salinity. To our knowledge, this is the first report on the characterization of an alpha-glucosidase from the halophilic Archaea, which could serve as a new model to gain insights into carbon metabolism in this understudied microbial group.

## 1. Introduction

*Haloquadratum walsbyi* is a squared-shaped, extremely halophilic member of the *Euryarchaeota*, which has been described as the dominant representative of the microbiota present in aquatic hypersaline (≥32% NaCl) environments [1,2,3]. The organism was first described in 1980 by Walsby [4]. However, subsequent studies employing conventional and molecular techniques have reported *Haloquadratum walsbyi* as the dominant lineage across a variety of environments with high osmotic stress including hypersaline pools [2,5], salt lakes, and saltern crystallizer ponds. Pure cultures of this archaeon were independently isolated from solar salterns in Spain and Australia by Bolhuis et al. [5] and Burns et al. [1], respectively. The Spanish strain was designated HBSQ001 and the Australian strain as C23. The species was formally described as *Hqr. walsbyi* in 2007 by Burns et al. [1]. *Hqr. walsbyi* grows optimally at 45 °C under strictly aerobic conditions in defined media (pH 7) with 18% (*w*/*v*) NaCl and supplemented with pyruvate as the only carbon source [1]. Nevertheless, as many as 43 genes with predicted functions related to carbohydrate metabolism, including a putative alpha-glucosidase encoded by an open reading frame (ORFs) *Hqrw_2071* and *HQ1911A*, have been described in both strains [5]. 

Alpha-glucosidases (EC. 3.2.1.20) are a diverse group of enzymes capable of hydrolyzing 1,4-alpha-glucosidic linkages of terminal residues of d-glucose in a variety of oligosaccharides [6,7]. They are known to carry out the release of glucose from maltose and maltodextrins and to mediate glycoprotein processing in living systems. Moreover, some of these enzymes are of biotechnological and industrial value since they can conjugate sugars with biologically useful materials and also facilitate the production of food-related oligosaccharides [8,9]. 

The first archaeal alpha-glucosidase was purified from *Sulfolobus solfataricus* strains 98/2 and P2 [10]. Subsequently, several alpha-glucosidases from members of the hyperthermophilic Archaea (*Sulfolobus shibatae* and *S. solfataricus*, *Pyrococcus furiosus*, *P. woesei*, and *Thermococcus litoralis*) have been have been characterized [11,12,13]. Several members of the halophilic archaea (mainly *Haloferax* and *Halogeometricum*) are capable of using alpha-linked sugars as carbon sources [8,9,14]. 

There are relatively few studies that deal with the purification and characterization of halophilic enzymes. Published examples include an extracellular serine protease produced by *Natrialba madagii* [15]; α-amylases from *Haloarcula hispanica* [16], *Haloferax mediterranei* [17], and *Halomonas meridiana* [18]; a glucose dehydrogenase from *Haloferax mediterranei* [19]; an alcohol dehydrogenase from *Natronomonas pharaonis* [20], and an extremely halophilic β-galactosidase from *Haloferax lucentense* [21,22]. However, to our knowledge, there are no reports on the characterization of an extremely halophilic alpha-glucosidase. In this work, we describe the cloning and characterization of an alpha-glucosidase gene from *Hqr. walsbyi* C23, the dominant haloarchaeon in most solar saltern systems according to metagenomic surveys [2].

## 2. Materials and Methods 

### 2.1. Microbial Strain

Cultures of *Haloquadratum walsbyi* type strain C23 (DSM 16854) were kindly provided by Dr. Mike Dyall-Smith (University of Melbourne, Melbourne, Australia). 

### 2.2. Construction of the Expression Plasmid

Genomic DNA from *Hqr. walsbyi C23* was extracted as previously described [23]. The resulting pellet from 2 mL of liquid culture was resuspended in 500 µL of lysis solution (sterile deionized water) and heated at 70 °C for 10 min. Primers flanking the *Hqrw_2071* locus in *Hqr. walsbyi* strain C23 were designed for the subsequent amplification, cloning and the expression of this gene. These primers were checked against the genome of strain HBQ001 and were also complementary to flanking regions of *HQ1911A*. Protein sequences predicted from both target loci were of equal length (782 amino acids), but differed by 10 amino acid substitutions. The putative alpha-glucosidase gene was PCR-amplified using TaKaRa La Taq DNA polymerase (Takara Bio, Mountain View, CA, USA). The sequences of forward and reverse primers containing *NheI* and *XhoI* recognition sites (underlined positions) were: 5′-CCA TAG CTA GCA TGT GGT TGG 3′ and 5′-CGT CTC GAG ACC TCA GGA AGT ATT GG-3′, respectively. The PCR product was cloned into pET28b(+) expression vector (Novagen, Madison, WI, USA), which was pre-digested with *NdeI* and *XhoI* (New England Biolabs Inc., Ipswich, MA, USA). The ligation was performed using T4 DNA Ligase (Promega Inc., Fitchburg, WI, USA). The resulting recombinant plasmid was called *pET-malH*.

### 2.3. Protein Expression and Purification

The recombinant plasmid (*pET-malH*) was transformed into *E. coli* Rosetta™ cells, and grown at 37 °C in 4 L of Luria Bertani broth (LB) containing 34 μg/mL chloramphenicol and 30 μg/mL kanamycin. When cultures reached late log phase (OD_600_ of 0.6–0.8), they were induced with 1 mM Isopropyl β-d-1-thiogalactopyranoside (IPTG) for 3 h. Cells were harvested by centrifugation (4000 rpm × 20 min, at 4 °C), resuspended in sodium phosphate buffer (NaH_2_PO_4_, pH 8.0; 3 M NaCl, 10 mM imidazole), and lysed by sonication on ice (100 W, 1 s of sonication vs. 2 s pause, 500 cycles). The cell lysate was then centrifuged (13,000 rpm, 15 min, 4 °C). The resulting supernatant was loaded into a chromatography column packed with Ni-NTA agarose (Qiagen, Venlo, Germany), and washed with sodium phosphate at increasing imidazole concentrations of up to 80 mM. The elution was performed using sodium phosphate containing 250 mM of imidazole. Eluted fractions were tested for alpha-glucosidase activity as described in Section 2.4. The alpha-glucosidase containing fractions were resolved by Polyacrylamide Gel Electrophoresis (SDS-PAGE) using 10% polyacrylamide gels, stained with Bio-Safe™ Coomasie Stain (BioRad Inc., Hercules, CA, USA). Protein concentration was determined using the Pierce BCA Protein Assay (ThermoScientific Inc., Bridgewater, NJ, USA), using bovine serum albumin (BSA) as a standard.

### 2.4. Enzymatic Assays

The alpha-glucosidase activity was determined by measuring the formation of *p*-nitrophenol (pNP) (OD at 420 nm, 40 °C) from the hydrolysis of *p*-nitrophenyl α-d-glucopyranoside (PNPG, a chromogenic α-glucosidase substrate) and 4-nitrophenyl α-d-xylopyranoside (PNPX, a chromogenic α-xylosidase substrate) [10,21,24]. The activity assays were initiated by adding samples of the crude extract or the purified enzyme to a reaction mixture consisting of 10 mM of each substrate, 50 mM 2-(*N*-morpholino)ethanesulfonic acid (MES), 3 M of KCl or NaCl (pH 6.0). Reactions were terminated after 30 min by the addition of 500 μL, 1 M Na_2_CO_3_.

### 2.5. Effects of Salinity, pH, and Temperature

The effect of salinity was determined using one-fold increments of KCl (0–4 M) and NaCl (0–5 M) in 50 mM MES (pH 6.0) at 40 °C. The optimal temperature was determined by carrying out assays incubated independently at 10, 25, 30, 40, 50, and 60 °C. The optimal pH was determined at 40 °C using the following buffers: citric acid (pH 2.5–3.5), sodium acetate (pH 4.0), MES (pH 5–6), Tris-HCl (pH 7–8), Na_2_HPO_4_ (pH 9.0), and *N*-cyclohexyl-3-aminopropanesulfonic acid (CAPS) (pH 10.0) supplemented with 3 M KCl. All enzymatic assays were performed at conditions described in Section 2.4.

### 2.6. Phylogenetic Analyses

Protein sequences of alpha-glucosidases from Archaea, Bacteria and Eukarya were retrieved from the Pfam protein family database [25], except that encoded by *Hqr. walsbyi* C23, which was obtained from the KEGG database [26]. Amino acid sequence alignments were generated using ClustalW [27] while and Neighbor-joining and maximum likelihood (data not shown) phylogenetic trees (2000 bootstrap replications) were constructed with MEGA 6 (http://www.megasoftware.net/)) [28,29,30] using the *p*-distance and JTT matrix-based models, respectively.

### 2.7. In Silico Functional Characterization of the Hqr. walsbyi Alpha-Glucosidase

The predicted amino acid sequence of the putative glycosyl hydrolase from *Hqrw_2071* was screened for the presence of functional domains using the NCBI’s CDD/SPARCLE Tools [31]. In order to detect functional features with respect to protein structure, the COBALT program [32] was used to generate an anchored multiple sequence alignment (MSA) based on functional constraints derived from 3D structure information contained in NCBI-curated domains. The alignment was generated by importing COBALT’s output into the Graphic View interphase of the BioEdit software package (http://www.mbio.ncsu.edu/BioEdit/page2.html) [27] and manually edited based on CDD annotations in a rich text file using a word processor and included sequences from archaeal alpha-glucosidases previously purified and characterized at the molecular level (Table 1). Additional searches for the presence of functional domains were conducted with the CDD tool using the Pfam and InterPro databases [25,26]. Moreover, structural models of the *Hqr. walsbyi* alpha-glucosidase were predicted using the Phyre2 www.sbg.bio.ic.ac.uk/~phyre/html/page.cgi?id=index) and (PS)2-V2 (ps2.life.nctu.edu) modelling servers. The resulting structures were visualized using the iCn3D web-based 3D structure viewer (https://www.ncbi.nlm.nih.gov/Structure/icn3d/full.html).

## 3. Results and Discussion

### 3.1. Identification of a Putative Alpha-Glucosidase Gene in the Hqr. walsbyi C23 Genome

Previous studies from our laboratory have demonstrated that *Hgm. borinquense* has the ability to utilize maltose as a sole carbon source. Phylogenetically, *Hqr. walsbyi* is closely related to *Hgm. borinquense* [1], and we hypothesized that these organisms might have a similar physiology for carbohydrate metabolism. Therefore, the genomes of *Hqr. walsbyi* HBSQ001 (DSM 16790) and *Hqr. walsbyi* C23 (DSM 16854) were searched for the presence of putative alpha-glucosidase gene sequences using the KEGG database (http://www.genome.jp/kegg/pathway.html). This resulted in the detection of a gene (*Hqrw_2071*) annotated as a putative alpha-glucosidase in the genome of *Hqr. walsbyi* strain C23 as well as in that of its homolog (*HQ1911A*) in strain HBSQ001. Through the use of different databases (Pfam, Expasy Proteomics Server, PROSITE, Inter Pro Scan, NCBI Conserved Domains), it was determined that the region comprised by nucleotide positions 1,126,713 to 1,129,061 of the *Hqr. walsbyi* C23 chromosome encoded an ORF with a predicted amino acid sequence of 782 residues [25,26,33]. The inferred amino acid sequence of *Hqrw_2071* was compared to that of other previously described or annotated alpha-glucosidases from members of the Archaea. Table 1 shows detected domains of *Hqrw_2071* from *Hqr. Walsbyi*, which are 35% identical with respect to those present among representatives of the Family 31 of the glycosyl hydrolases and the family of galactose mutarotase-like 2. Galactose mutarotases act as catalyzers in the interconversion of either α- and β-anomers of galactose to glucose [34].

### 3.2. Biochemical Characterization of the Recombinant Alpha-Glucosidase from Hqr. walsbyi

After IPTG induction, crude extracts from *Escherichia coli* cells were analysed for the detection of alpha-glucosidase activity at salinity concentrations ranging from 0 to 5 M. Crude extracts from cells containing the pET-*mal*H plasmid showed alpha-glucoside activity in assays carried out at 40 °C and supplemented with 3 M NaCl. In contrast, extracts from cells transformed with an empty vector or from cells with no vector were unreactive (data not shown). 

**Figure 1:**
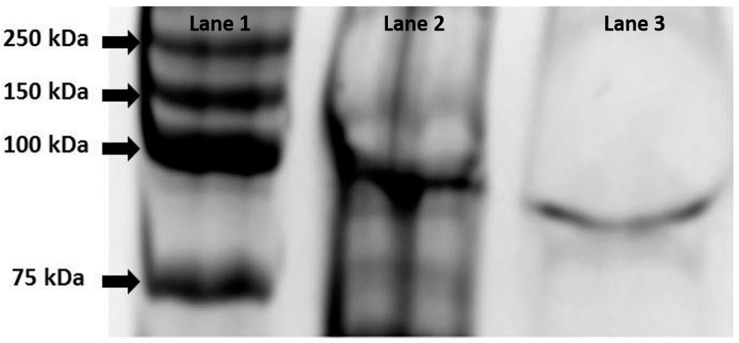
SDS-PAGE gel (10% polyacrylamide) of α-glucosidase containing fractions. Lane 1: Ladder Precision Plus Protein Kaleidoscope (BioRad Inc., Hercules, CA, USA); Lane 2: Crude extract of *E. coli* Rosetta™ cells (*pET-malH*) after induction with IPTG; Lane 3: purified MalH showing a size of approximately 87 kDa.

Purification of the recombinant alpha-glucosidase was performed by loading 1 mL of concentrated crude cell extract into a Ni-NTA agarose column (Qiagen, Venlo, Germany) and eluted with imidazole as described by the manufacturer. The quality and purity of the recombinant protein was verified using SDS-PAGE (Figure 1). 

**Figure 2:**
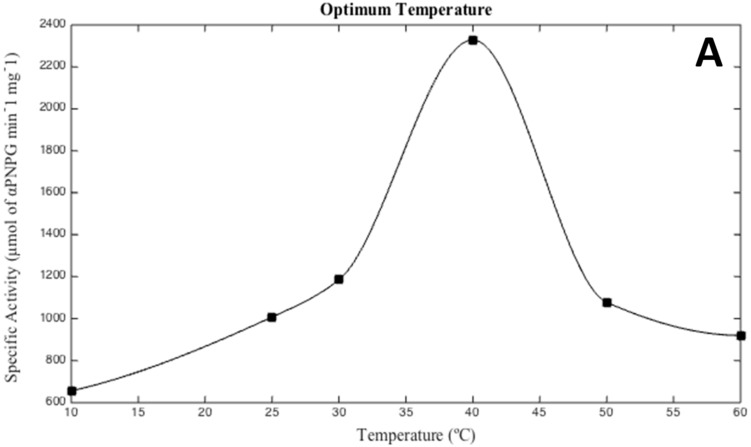

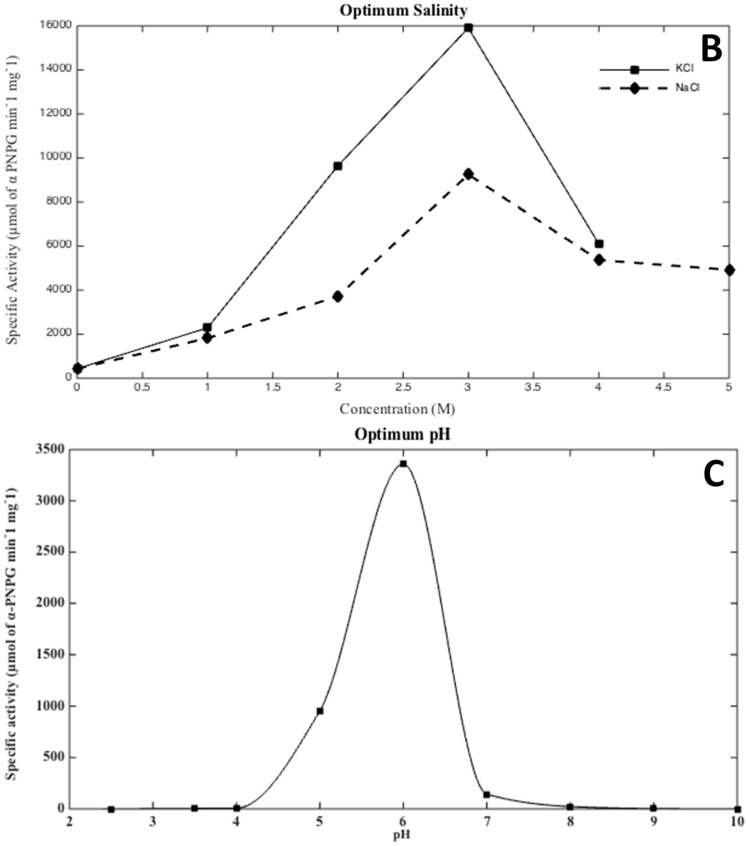
Optimal temperature (**A**), salinity (**B**), and pH (**C**) conditions for the hydrolysis of p-nitrophenyl α-d-glucopyranoside (PNPG) by the *Hqr. walsbyi* MalH. Conditions were tested as follows: (**A**) optimal temperature was determined using the following: 10, 25, 30, 40, 50, 60 (°C) in a 3 M KCl, 50 mM MES (pH 6.0) reaction buffer; (**B**) optimal salinity was determined using KCl (0–4 M) and NaCl (0–5 M) as salt variables in a reaction buffer containing 50 mM MES (pH 6.0) at 40 °C; (**C**) optimum pH was determined at 40 °C with 3 M KCl in the following range: 2.5, 3.5, 4.0, 5.0, 6.0, 7.0, 8.0, 9.0, and 10.0. The graphs are representative of an average of four independent trials performed in order to describe the optimal conditions of the protein under study.

As seen in Figure 2, optimal activity conditions for the recombinant enzyme were observed at 40 °C, pH 6.0, and 3 M KCl. These results are in agreement with *Hqr. walsbyi* growth conditions [1]. Interestingly, recombinant MalH showed a higher activity when KCl was used in the buffer instead of NaCl. This result is consistent with a cytoplasmic enzyme, as halophilic Archaea accumulate high levels of K^+^ in their cytoplasm to compensate for the high concentration of Na^+^ in their environment [39].

### 3.3. In Silico Functional Chracterization and Phylogenetic Analysis of the Hqrw_2071 Gene Product

**Figure 3:**
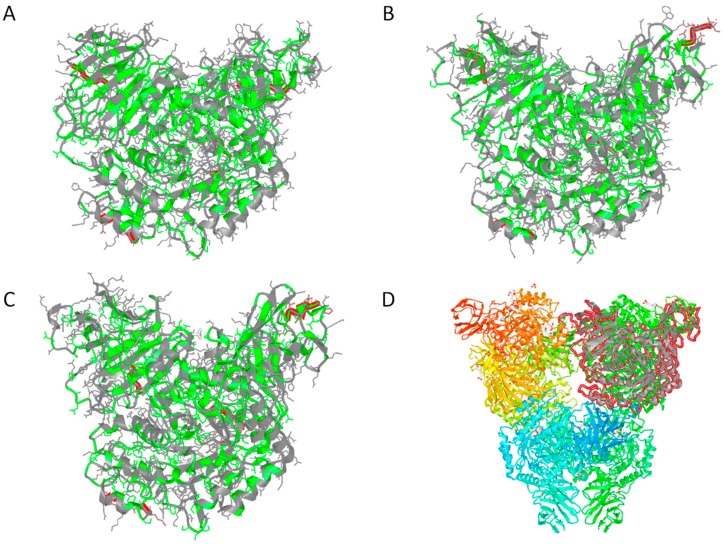
Tridimensional structural predictions for Hqr. walsbyi MalH generated with the Phyre2 (**A**) and (PS)2-V2 (**B**) modellingmodeling tools; (**C**) tridimensional structure of the monomer corresponding to the chain A of the homohexadimer complex of the *E. coli* YicI-like alpha-xylosidase (PDB 2F2H). Red highlights depict the first five positions of the N-terminal and the last five residues of the C-terminal (left and right, respectively) as well as that of putative homotrimer and hexadimer interfaces (bottom and center, respectively). Hydrophobic and hydrophilic residues are highlighted in green and gray, respectively (**A**–**C**); (**D**) Homohexadimer structure of the YicI thiosugar Michaelis complex (PDB 2F2H). The monomer corresponding to the chain A of the complex is shown in gray and outlined in red (top right). Secondary structure domains within each monomer are highlighted in different shades.

With regard to global relationships, structurally-constrained sequence alignments revealed that MalH was nearly 50% identical to the partial sequence of an halophilic glycosidase detected in the metagenome of an Australian hypersaline lake [40] and aproximately 36% identical to putative alpha-glucosidases from *H. kocurii* and *H. litoreum* [41]. Despite the detection of functional traits shared with xylosidases, MalH only shared 26% amino acid identity with respect to its closest database match with a similar domain architecture, an *E. coli* YicI alpha-xylosidase [42]. However, similar to theYicI alpha-xylosidases (PDB 2F2H), MalH seems capable of forming homo-multimers since residues potentially associated with binding of homotrimers (T353, G352, and E361) and homohexamers (R485, F488 and E497) were detected using the CDD tool. These findings were in agreement with 3D structure prediction analyses generated by the Phyre2 server, which suggested a folding pattern consistent with that of various alpha-glucosidases (100% of residues modeled at >90% confidence) in which most of the hydrophobic residues were oriented towards the core of the predicted structure (Figure 3A). Likewise, the 3D model produced by the (PS)2-V2 server revealed strong structural similarities (E-value 4.2 × 10^–28^) with respect to a homo-multimeric YicI alpha-xylosidase from *E. coli* (PDB: 1WE5 and 2F2H; Figure 3B–D). Furthermore, the (PS)2-V2 server aligned 98% of the amino acid sequence of MalH with the PDB 2F2H-derived template at a 25.89% of amino acid identity. An identity value similar to that obtained from structurally constrained alignments (26%) using the COBALT tool.

**Figure 4:**
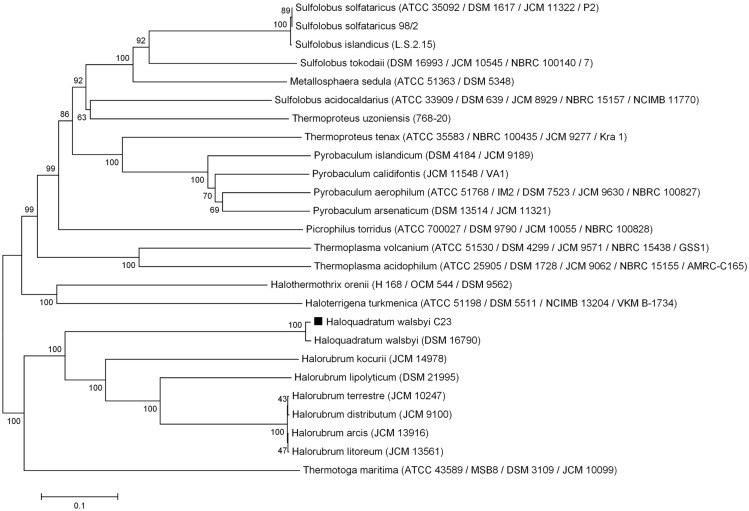
Neighbor-joining dendrogram showing the evolutionary relationship of archaeal alpha-glucosidases [44]. The Thermotoga maritima alpha-glucosidase was used as an outgroup. The sequence of *Hqr. walsbyi* MalH is labeled with a black square. Bootstrap values (2000 replicates) are shown at the nodes [27]. The phylogenetic tree is drawn to scale, using the same units for branch lengths as those of the evolutionary distances. The evolutionary distances were estimated using the p-distance model and are in the units of the number of amino acid differences per site [25]. The analysis involved 26 protein sequences. A total of 998 positions were used in the dataset.

**Figure 5:**
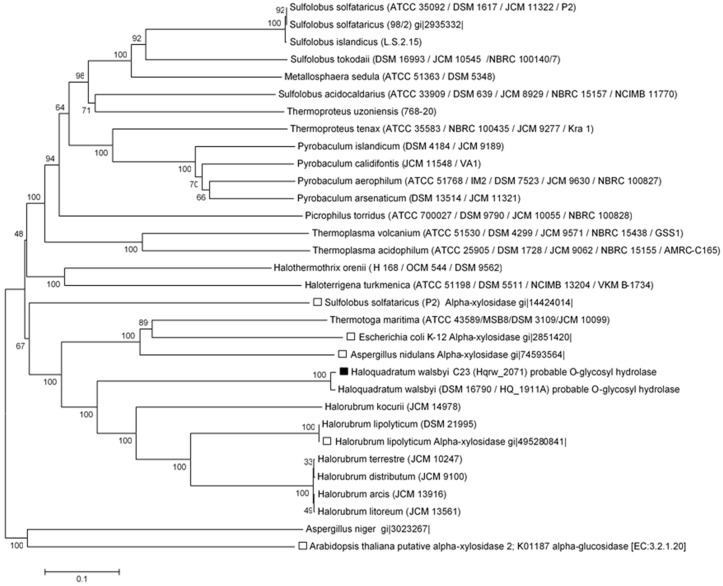
Inferred evolutionary relationships of α-glucosidases and alpha-xylosidases described among representatives from the Archaea, Bacteria, and Eukarya. The position of MalH is indicated by the black square, while that of putative alpha-xylosidases is indicated with white squares. Alpha-glucosidases are unmarked. The evolutionary history was predicted using the neighbor-joining method [28]. The percentage of replicate trees in which the associated taxa clustered together in the bootstrap test (2000 replicates) are shown at the nodes [27]. The phylogenetic tree is drawn to scale, using the same units for branch lengths as those of the evolutionary distances. Evolutionary distances were calculated using the *p*-distance method and are in the units of the number of amino acid differences per site. The analysis included 32 amino acid sequences. All ambiguous positions were removed for each sequence pair. The final dataset consisted of a total of 2541 positions. Evolutionary analyses were conducted in MEGA 6 [29].

Phylogenetic tree reconstructions showed that the *Hqr. walsbyi* MalH is ≤50% identical to other alpha-glucosidases within the Archaea (Table 1, Figure 4 and Figure 5, Figure S1) although similarities with other glycosyl hydrolases, such as alpha-xylosidases, were observed through multiple sequence alignments. However, the recombinant protein from this study was unable to hydrolize α-PNPX (data not shown). It is suggested that genes encoding xylosidases and glucosidases are homologs, but evolutionary changes could have separated them and, therefore, both types of enzymes show degrees of similarity when compared in phylogenetic trees [7,43]. 

In silico analysis showed two putative transmembrane helices and four putative transmembrane segments. Putative transmembrane helices are also found in eukaryotic alpha-glucosidases. However, since enzymes from eukaryotes do not span the cytoplasmic membrane, the predicted transmembrane segments of the *malH* product could be involved in enzyme folding rather than membrane attachment [43]. 

**Figure 6:**
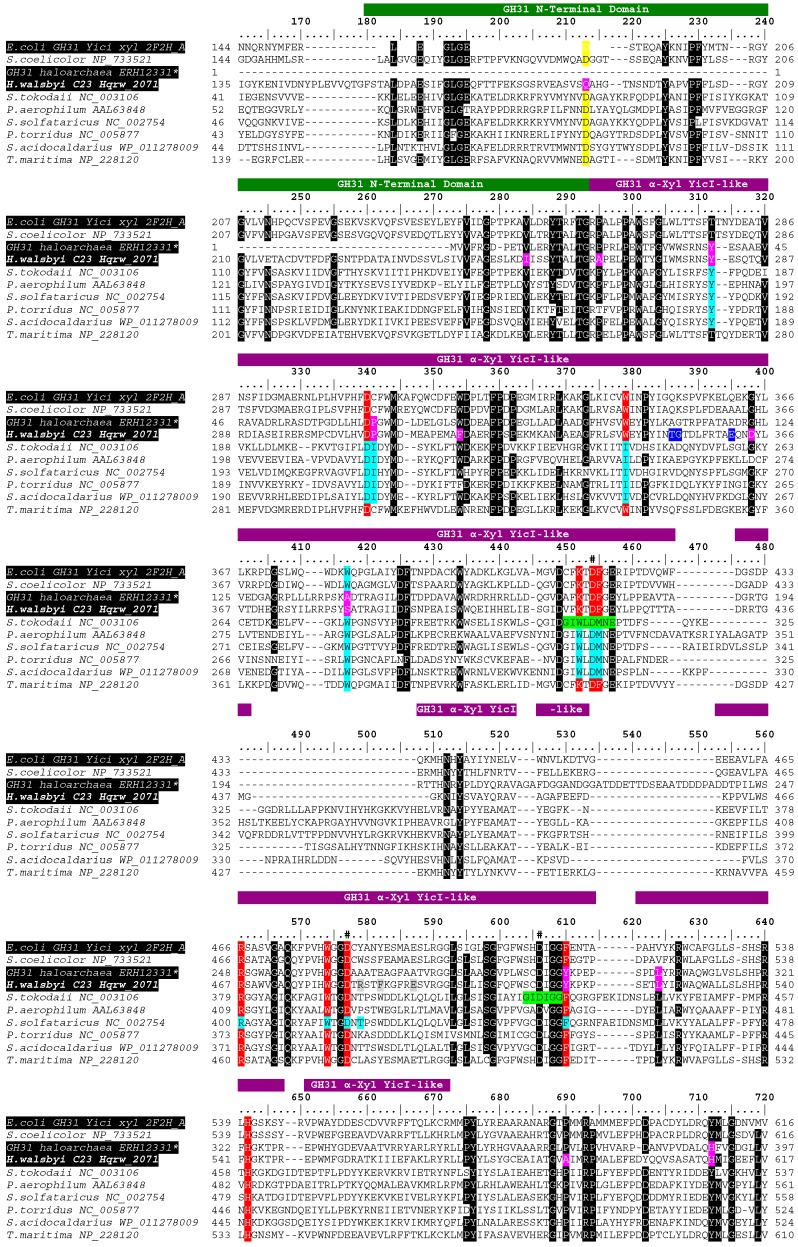
Partial depiction of a full-length multiple sequence alignment showing the functional features detected in MalH, with respect to those described in previously purified and characterized alpha-glucosidases. The green bar indicates the potential location for an N-terminal domain of GH31 enzymes, whereas the violet bar shows thatfor a YicI-like GH31 xylosidase domain. Black-shaded columns illustrate positions ≥75% identical. Yellow and red columns indicate the location of active sites detected at regions corresponding to the putative N-terminal and YicI-like domains, respectively. Light blue and light green shades show positions corresponding to active sites and conserved regions reported in glycosidases from *S. solfataricus* and *S. tokodaii*, respectively. The location of putative catalytic “D” residues associated with YicI-like domains are indicated with “#” characters at the top of the column. That of predicted homotrimer and homohexamer binding sites are highlighted in blue and gray, respectively. Distinctive residues detected in the predicted gene product of *Hqrw_2071* are highlighted in pink. PDB or GenBank accession numbers follow the designation of sequences. * = partial sequence.

Functional predictions based on analyses performed with the NCBI’s Conserved Domain Database (CDD) and the Subfamily Protein Architecture Labeling Engine (SPARCLE) revealed a domain configuration reminiscent of an alpha-xylosidase (Arch. ID 1020107). This consisted of a conserved N-terminal domain of the glycoside hydrolase family 31 (GH31_N) (E-value 5.80 × 10^−31^) located upstream with respect to a YicI-like domain of GH31 xylosidases (E-value 3.27 × 10^−149^). These were identified at intervals 154–262 and 262–570, respectively (Figure 6; MalH numbering system). 

**Figure 7:**
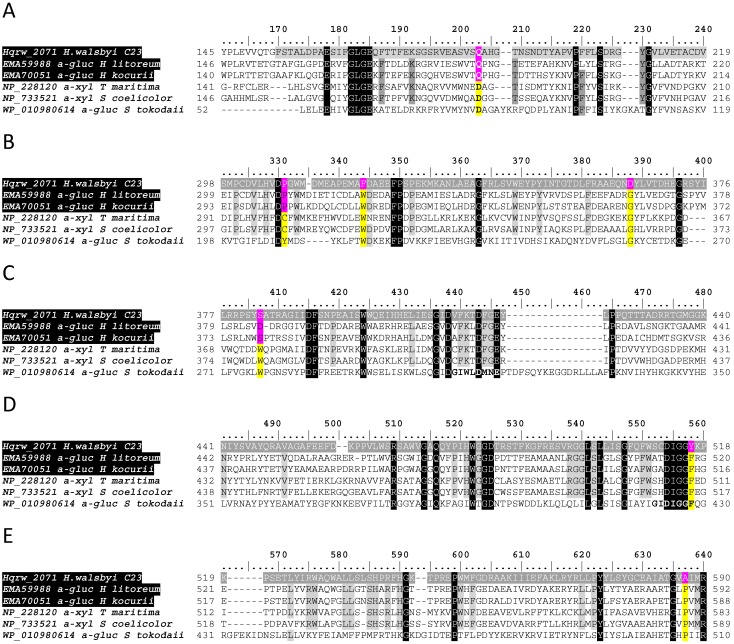
Partial multiple sequence alignments (**A**–**E**) illustrating distinctive positions (pink) shared by glucosidases from halophilic archaea with respect to conserved sites in their bacterial (*S. coelicolor*) and thermophilic archaeal counterparts (yellow). Gray-shaded rows indicate the predicted span the N-terminal domain of GH31 glycosyl hydrolases (**A**) located upstream with respect to a YicI-like xylosidase domain (**B**–**E**). Black-shaded columns illustrate the position amino acids as 100% identical, whereas light gray columns indicate the location of residues ≥84% identical. GenBank accession numbers precede the designation of reference sequences.

A conserved glutamine residue (Q187; Figure 7) previously described as part of an active site in the tridimensional structures of the N-terminal domain of GH31 alpha-glucosidases (cd14752) from the bacterium *Ruminococcus obeum* PDB: 3PHA [45] and common beet, *Beta vulgaris* PDB: 3W37 [46] was detected. However, Q187 emerged as all the other functionally confirmed alpha-glucosidases compared in this alignment had aspartic acid (D213) at this position (Figure 6). These included enzymes for bacteria and thermophilic archaea including in the conceptual translation of an alpha-xylosidase from the reference genome of *Streptomyces coelicolor* (GenBank accession No. NP_733521) and in that of a YicI-like alpha-xylosidase from *E. coli* (PDB: 2F2H_A) [42]. Moreover, the Q187 substitution was an exclusive trait of MalH as well as of sequences provisionally identified as alpha-glucosidases in *Halorubrum litoreum* and *Halorubrum kocurii* [33,41], suggesting a signature feature of the N-terminal domain of alpha glucosidases from halophilic archaea (Figure 7A). All residues (D307, W445, K417, D419, F420, R467, W480, Y516 and H552; Figure 6, MalH numbering system; red-colored positions) comprising the active site and the two catalytic residues (D419 and D483) responsible for the hydrolysis reaction in the YicI-like alpha-xylosidase from *E. coli* (PDB: 2F2H_A) [42] were also identified in MalH (Figure 6). Further analyses using the Pfam database [25] showed the presence of domains with functions and coordinates consistent with those detected using the CDD tool. These consisted of a galactose mutarotase-like domain at the N-terminal ([43]; E-value 4.9 × 10^−12^) followed by a GH31 domain (E-value 1.4 × 10^−103^), which were situated at intervals 162–222 and 244–670, respectively. 

Four of the 11 active sites described in the tridimensional structure of the *Sulfolobus solfataricus* alpha-glucosidase MalA (PDB: 2G3N) were identified in MalH (Figure 6, light blue highlights). Moreover, MalH and *S. solfataricus* MalA also shared the two catalytic D residues characteristic of GH31 affiliates, which were also present in a thermostable alpha-glucosidase from *Sulfolobus tokodaii* (Figure 6, Green highlights) [37]. Nevertheless, MalH displayed a variety of unique substitutions at functional or conserved sites (Figure 6 and Figure 7, pink highlight) that appeared as distinguishing features from enzymes originating from bacterial and thermophilic archaea. Furthermore, in some instances, the same substitutions were shared among putative glycosidases from halophilic hosts, further strengthening the presence of signature traits for this group of proteins (Figure 7, pink residues). 

In general, the domain-constrained comparative alignment revealed distinctive substitutions that could account for the inability of the MalH to produce xylose from αPNPX and its high activity on αPNPG. The functionality of representative from the GH31 family is known to be diverse, as this group is comprised of enzymes having functions that include alpha-galactosidase, alpha-glucosidase, alpha-xylosidase, glucoamilase, sucrase-isomaltase, and α-glucan lyase activities [47,48]. Moreover, several GH31 are known to exhibit both glycosidadse and xylosidase activities with different levels of affinity between these substrates [7,49,50]. 

The importance of halophilic enzymes has been reviewed elsewhere, and is not limited only to food processing, bioremediation, and biosynthesis [8,43,47,48,50,51,52]. Specifically, glucosidases are studied for their potential in multiple industry processes due to their thermostability [48]—for example, they can be used for the production of biofuels and pharmaceutical products, for enhancing the wine aroma, and for reducing the toxic compounds present in animal feed [48]. The stability of glucosidases under high salt conditions remains poorly understood, and this study provides an example of a novel alpha-glucosidase (MalH) with unique characteristics from the halophilic archaea that could be used to address this gap. MalH might provide insights about glucosidase activity under high salinity conditions. To our knowledge, this is the first report of the cloning and molecular characterization of a novel alpha glucosidase with high salinity requirements, which can help study carbon utilization of *Haloquadratum* in hypersaline environments.

## Figures and Tables

**Table 1:** Archaeal members encoding hydrolases with conserved domains associated with alpha-glucosidases. The identity percentages of these conserved regions with respect to those detected in the amino acid sequence of the *Haloquadratum walsbyi* MalH are shown. The comparisons are based on searches against the Pfam repository.

Archaea Species	Conserved Domains		Percent Identity (%)
	Galactose Mutarotase-Like 2	Glycosyl Hydrolases Family 31	
*Haloquadratum walsbyi*	161–222	243–670	This study
*Halorubrum kocurii*	156–217	238–668	43
*Halorubrum terrestre*	162–223	244–672	41
*Halorubrum arcis*	162–223	244–672	41
*Halorubrum litoreum*	162–233	244–672	41
*Halorubrum distributum*	162–233	244–672	41
*Halorubrum lipolyticum*	160–221	242–670	40
*Halothermothrix orenii*	144–211	232–672	29
*Thermoplasma volcanium*	177–244	265–697	27
*Thermoproteus uzoniensis*	67–136	157–602	27
Pyrobaculum aerophilum *	67–133	152–612	27
*Haloterrigena turkmenica*	126–193	226–696	26
*Sulfolobus islandicus*	61–127	148–608	26
Sulfolobus solfataricus *	161–222	243–670	26
*Thermoproteus tenax*	69–135	155–616	26
*Pyrobaculum arsenaticum*	68–134	154–613	26
*Pyrobaculum calidifontis*	67–133	153–610	26
*Thermoplasma acidophilum*	147–214	234–667	25
*Sulfolobus acidocaldarius*	59–124	145–574	25
Sulfolobus tokodaii *	56–122	143–586	25
*Metallosphaera sedula*	57–123	144–598	25
*Pyrobaculum islandicum*	67–133	153–612	25
Picrophilus torridus *	58–123	144–572	24

* Molecularly characterized and purified [35,36,37,38].

## Supplementary Materials

The following are available online at www.mdpi.com/2075-1729/7/4/46/s1, Figure S1: Evolutionary relationship of alpha-glucosidases within Archaea by Maximum Likelihood method.
