# Supplementary material for: Cloning and Molecular Characterization of an Alpha-Glucosidase (MalH) from the Halophilic Archaeon Haloquadratum walsbyi
Source: Life (Basel). 2017 Nov 21;7(4):46. doi: 10.3390/life7040046 (PMC5745559; doi:10.3390/life7040046)
Supplement: Supplementary file 1 [file life-07-00046-s001.pdf]

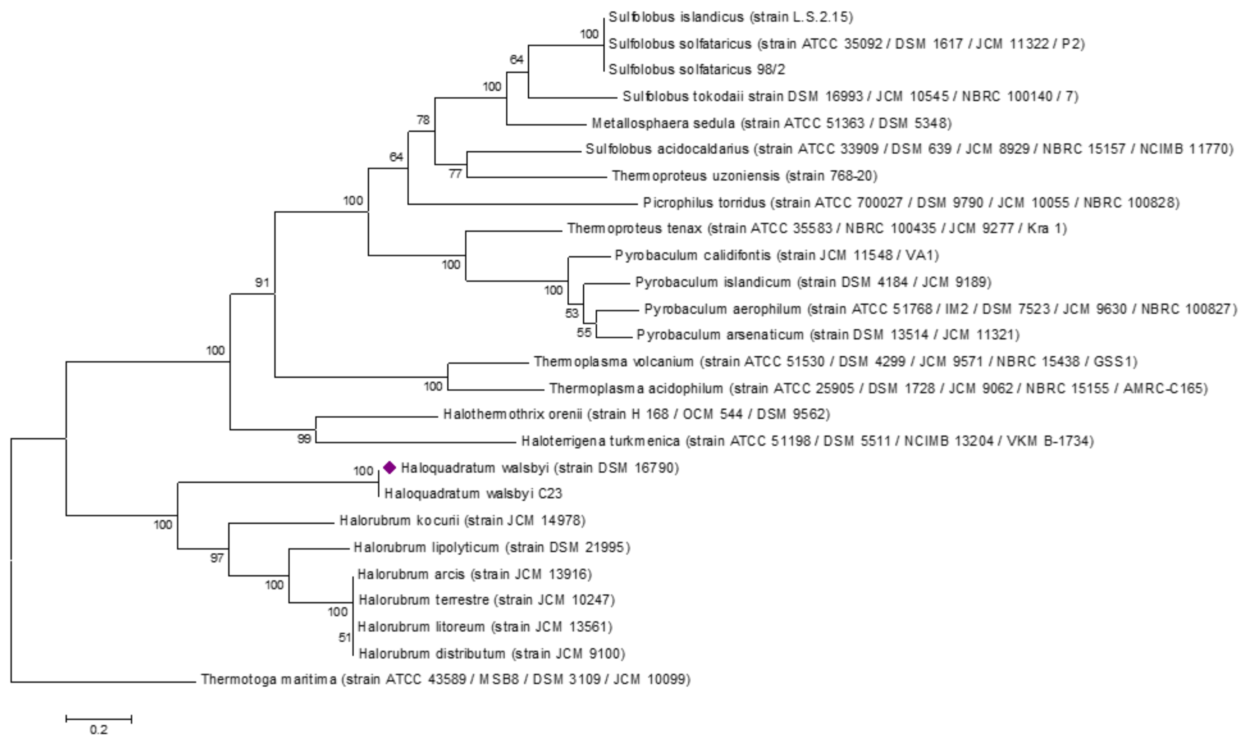

Supplemental Figure 1. Evolutionary relationship of alpha-glucosidases within Archaea by Maximum Likelihood method. The method was used after an alignment with ClustalW for the analysis of phylogenetic relationships of 26 aminoacid sequences coding for alpha-glucosidases from different Archaea members. JTT matrix-based model was used as base of Maximum Likelihood method. Taxa clustered together was determined by the percentage next to the branches. Initial tree was obtained by the application of Neighbor-join and BioNJ algorithms to a matrix of pairwise distances estimated by JTT model and further selecting topology with the best log likelihood value (-18433.5445). The tree is drawn to scale, with branch lengths measured in the number of substitutions per site. All positions with less than 100% site coverage were eliminated. That is, fewer than 0% alignment gaps, missing data, and ambiguous bases were allowed at any position. A total of 533 positions were found in the final dataset. Evolutionary analyses were conducted in MEGA6 [26].
